# The complete chloroplast genome sequence of *Melliodendron wangianum* (Styracaceae)

**DOI:** 10.1080/23802359.2019.1688707

**Published:** 2019-11-13

**Authors:** Yabo Wang, Xiaogang Xu, Lili Tong, Yaoqin Zhang, Zixun Zhao

**Affiliations:** a College of Biology and Environment, Nanjing Forestry University, Nanjing, China;; b Co-Innovation Center for Sustainable Forestry in Southern China, Nanjing Forestry University, Nanjing, China;; c School of Horticulture & Landscape Architecture, Jinling Institute of Technology, Nanjing, China

**Keywords:** *Melliodendron wangianum*, styracaceae family, chloroplast genome, Phylogenomics

## Abstract

*Melliodendron wangianum* Hu (Styracaceae) is a rare endemic tree of Styracaceae in China. In this work, we set forth the complete chloroplast (cp) genome sequence of *M. wangianum* using next-generation sequencing. The entire cp genome was determined to be 157,196 bp in length. It contained large single-copy (LSC) and small single-copy (SSC) regions of 90,210 bp and 18,4561 bp, respectively, which were separated by a pair of 24,265 bp inverted repeat (IR) regions. The genome contained 128 genes, including 83 protein-coding genes, 37 tRNA genes, and eight rRNA genes. The overall GC content of the genome is 37.19%. Phylogenetic analysis confirmed the traditional family-level Taxonomy of *Melliodendron*, indicating that *M. wangianum* is closely related to a sister species to *M. xylocarpum* in Styracaceae.

*Melliodendron wangianum* Hu is an endemic species to China. *M. wangianum* is a member of *Melliodendron,* but it is listed as a synonym of *M. xylocarpum* (Grimshaw and Rix 2013). It is a multipurpose economic arbor species integrating ornamental, oil and timber values. The complete genome sequence of *M. wangianum* plays an important role in the protection, development and utilization of its resources. Here, we characterized the complete cp genome sequence of *M. wangianum* (GeneBank accession number: MN378563) based on Illumina pair-end sequencing to provide a valuable complete cp genomic resource.

The total genomic DNA was isolated from the fresh leaves of *M. wangianum* natively habitated in Lan-chi-tzu (32.1566 N, 118.9690 E) in Ma-pien Hsien, Sichuan, China. The voucher specimen was kept in the herbarium of Nanjing Forestry University (accession number: NF2017169). The whole genome sequencing was carried out on Illumina Hiseq platform by Nanjing Genepioneer Biotechnology Inc. (Nanjing, China). The original reading was filtered by CLC Genomics Workbench v9, and the clean reading was assembled into chloroplast genome with SPAdes (Bankevich et al. 2012). Finally, CpGAVAS (Liu et al. 2012) was used to annotate the gene structure and OGDRAW (Lohse et al. 2013) was used to generate the physical map. Based on the maximum likelihood (ML), the phylogenetic tree was deduced by MAFFT (Katoh and Standley 2013). The plastome of *M. wangianum* was determined to comprise double stranded, circular DNA of 157,196 bp containing two inverted repeat (IRa and IRb) regions of 24,265 bp each, separated by large single-copy (LSC) and small single-copy (SSC) regions of 90,210 bp and 18,4561 bp, respectively (NCBI acc. no. MN378563). The genome contained 128 genes, including 83 protein-coding genes, 37 tRNA genes, and eight rRNA genes. The five protein-coding genes, seven tRNA genes and four rRNA genes were duplicated in IR region. Twelve genes contained one intron and two genes (*clpP* and *ycf3*) contained two introns. The over-all GC content of *M. wangianum* cp genome is 37.19% and the corresponding values in LSC, SSC and IR regions are 35.3%, 30.57%, and 43.21%%, respectively.

To explore the taxonomic status of *M. wangianum*, the sequences of 28 chloroplast genomes (20 Styracaceae, 2 Symplocaceae, 2 Actinidiaceae and 4 Theaceae) were compared, and the maximum likelihood was constructed by fast tree version. The maximum likelihood evolutionary tree shows that *M. wangianum* is most relevant to *M. xylocarpum*, with bootstrap support value of 100% (Figure 1).

**Figure 1.**
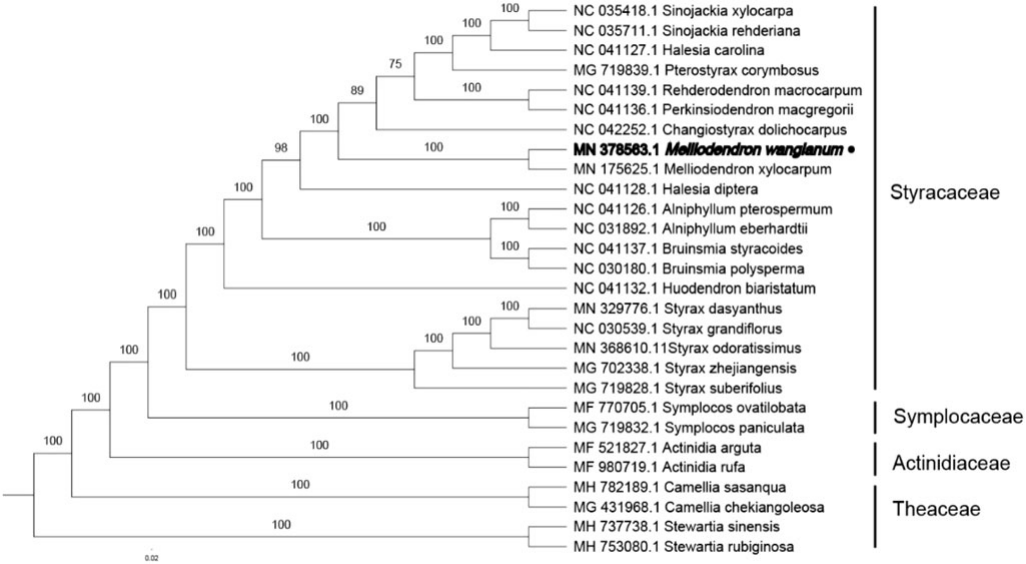
Phylogenetic tree inferred by maximum-likelihood (ML) method based on the complete chloroplast genome of 28 representative species. The bootstrap support values are shown at the branches.
